# Ultrasound evaluation of common carotid artery blood flow in the Labrador retriever

**DOI:** 10.1186/1746-6148-9-195

**Published:** 2013-10-07

**Authors:** Denis J Svicero, Danuta P Doiche, Maria J Mamprim, Marta C T Heckler, Rogério M Amorim

**Affiliations:** 1 School of Veterinary Medicine and Animal Science, São Paulo State University, UNESP, Botucatu, Sao Paulo 18618-970, Brazil

**Keywords:** Doppler ultrasonography, Common carotid artery, Blood flow, Velocity, Dogs

## Abstract

**Background:**

Doppler ultrasound (DUS) examination provides quantitative and qualitative information concerning the blood flow in veins and arteries, enabling their morphological evaluation and the collection of hemodynamic data. Dogs and cats as well as humans may display neurological signs of brain hypoperfusion secondary to common carotid alterations. Hence, DUS examination might aid in the differential diagnosis of neurological disorders of ischemic origin, among other causes. The objective of this study was to register normal values for systolic peak velocity, minimum diastolic velocity, diameter and resistance index of both common carotid arteries of 12 healthy Labrador retriever dogs between 2 and 5 years of age. By gathering these values, we might be able to improve the sensitivity of hemodynamic studies in clinically important brain disorders.

**Results:**

There were no statistical differences between the values for the right and left vessels: the systolic peak velocity was 75.8 ± 16 cm/s, minimum diastolic velocity was 12.2 ± 4 cm/s, common carotid diameter was 0.545 ± 0.063 cm, and resistance index was 0.83 ± 0.07.

**Conclusions:**

The results of this study might be used to establish normal parameters for Labrador retriever dogs and thus help in the diagnosis of neurological disorders associated with alterations of the carotid arteries. Similar studies must be performed to evaluate the same parameters in other dog breeds of different sizes and skull conformations.

## Background

Doppler ultrasound (DUS) provides images of large veins and arteries throughout the whole body of animals. It not only evaluates their morphology, but also provides hemodynamic information of the main abdominal and peripheral vessels [1]. DUS has been used in veterinary medicine since the late 1980s. Research has been carried out to set standards for normal blood flow in the portal veins of dogs and evaluate the possible changes in these veins in chronic liver disorders [2]. DUS vascular examination must follow a predetermined study protocol that may vary according to the vessel to be analyzed, individual variances, preparation of the animal, animal behavior, obesity, and other factors [1].

In general, vascular DUS is performed in a bidimensional mode. Longitudinal and transverse images are obtained, and the echographic aspects of wall thickness, luminal content, luminal diameter, and vessel reaction to the pressure applied by the transducer are observed. The Doppler study provides information about vessel patency as well as blood flow direction and velocity. Spectral tracing allows for the study of Doppler wave morphology and documentation of possible pathologic findings [1].

Both left and right common carotid arteries in cats and dogs stem from the brachiocephalic trunk (first branch of the aortic arch). From there, they give off small branches such as the caudal and cranial thyroid arteries, and only then give rise to its terminal branches, the internal and external carotids of both sides [3]. The carotid sinus is identified as a distention in the internal carotid bulb. A relevant characteristic of this artery is the fact that it does not give rise to ramifications in its extracranial route going up through the lateral surface of the larynx [3]. The external carotid artery gives off several branches until it divides into the superficial temporal artery and maxillary artery. Among these branches are the occipital arteries, cranial laryngeal artery, lingual artery, facial artery, and caudal auricular artery [3].

In human medicine, cerebrovascular disorders are frequent and are very often related to atherosclerotic plaques and carotid artery stenosis. People with neurological signs are often subjected to carotid artery DUS examination. For this reason, thorough examination of such arteries has become a regular practice in the prevention of stroke, brain ischemia, and dementia [4]. A decline in cognitive function from ischemic, hypoperfusive, or hemorrhagic brain lesions is termed vascular cognitive impairment [5] and has been associated with cognitive decline in aging and Alzheimer’s disease [6]. Evidence for the coexistence of cognitive injury in patients with neurologic deficits from carotid stenosis is quite forthcoming [5].

In cats and dogs, atherosclerotic disorders do not occur with the same frequency as in humans. However, stenosis may occur because of other causes such as neoplasia (in the arteries as well as in the head and neck region, resulting in vascular involvement), aneurysms, arteriovenous malformations, trauma, and iatrogenic causes secondary to surgical procedures [7-9].

Based on human reports [4,10], we can speculate that dogs might manifest neurological signs due to carotid artery changes that lead to cerebral hypoperfusion. However, studies have not been performed in veterinary medicine to establish the relationship between common carotid artery disease and neurological signs, especially in dogs and cats. DUS examination is applicable to the diagnosis of artery disorders, particularly because it is a noninvasive examination of relatively low cost that does not require anesthetic procedures.

Normal blood flow in the carotid arteries of small animals may show wide variations. In general, larger dog breeds present bidirectional blood flow, whereas smaller dog breeds and cats present unidirectional blood flow. In 2004, Lee et al. observed an average systolic peak velocity of 115 ± 17 cm/s and an average final diastolic velocity of 39 ± 7 cm/s in the common carotid artery of 1-year-old beagles. They observed that the caudal velocity was generally higher than the cranial velocity [9,11].

In humans, when the internal and external carotid arteries are within normal standards, they can be singled out based on their spectral traces; both the internal and common carotid arteries present low-resistance flow, whereas the external carotid artery presents high-resistance flow [12]. However, some differences in the ultrasound parameters of elderly patients have been observed in human medicine [13]. These changes may also occur in elderly canine and feline patients, and further studies are necessary to clarify such events.

There is an information gap with respect to measurements and velocity data of the internal and external common carotid arteries of dogs and cats of different breeds and sizes. Obtaining this information will improve vascular disorder diagnostics in such species. The objective of our study was to measure the systolic peak velocity, minimum diastolic velocity, diameter and resistance index of both common carotid arteries of 12 healthy Labrador retrievers aged between 2 and 5 years. Obtaining such values may increase the sensitivity of hemodynamic studies of clinically important brain disorders.

## Results and discussion

No statistical differences were found between genders or carotid artery sides for minimum diastolic velocity (p = 0.838 and 0.193, respectively), systolic peak velocity (p = 0.256 and 0.885, respectively), vessel diameter (p = 0.216 and 0.590, respectively), pulsatility index (p = 0.671 and 0.356, respectively), or resistance index (p = 0.595 and 0.356, respectively). Nevertheless, individual results are shown in Table 1.

**Table 1:** Individual results

**Animal**		**Females**						**Males**					
		**1**	**2**	**3**	**4**	**5**	**6**	**7**	**8**	**9**	**10**	**11**	**12**
R. Car. Art.	Diastolic Min. V.	14	11	14	8	7	8	11	9	10	11	12	16
	Systolic. Max. V.	55	84	70	82	70	72	65	117	72	72	73	72
	Diameter (cm)	0.41	0.54	0.53	0.59	0.5	0.53	0.56	0.63	0.43	0.55	0.6	0.55
	PI	1.19	1.54	1.33	1.64	1.64	1.60	1.42	1.71	1.51	1.47	1.44	1.27
	RI	0.75	0.87	0.80	0.90	0.90	0.88	0.83	0.92	0.86	0.85	0.84	0.78
		**1**	**2**	**3**	**4**	**5**	**6**	**7**	**8**	**9**	**10**	**11**	**12**
L. Car. Art.	Diastolic Min. V.	12	14	10	24	8	12	15	12	16	7	17	10
	Systolic. Max. V.	65	115	64	56	63	66	87	101	80	87	68	64
	Diameter (cm)	0.42	0.52	0.54	0.6	0.58	0.54	0.56	0.68	0.5	0.52	0.64	0.5
	PI	1.38	1.57	1.46	0.80	1.55	1.38	1.41	1.58	1.33	1.70	1.20	1.46
	RI	0.82	0.88	0.84	0.57	0.87	0.82	0.83	0.88	0.80	0.92	0.75	0.84

R. Car. Art: right carotid artery; L. Car. Art: left carotid artery; Min. V: minimum velocity (cm/s); Max. V: maximum velocity (cm/s); PI: pulsatility index; RI: resistance index.

The average, standard deviation, and variation coefficient for the minimum diastolic velocity, systolic peak velocity, vessel diameter, pulsatility index and resistance index of both the right and left common carotid arteries and their respective genders are shown in Table 2.

**Table 2:** Spectral waveform analysis and diameter for normal profile in dogs divided by gender and side

**Animal**		**Females**		**Males**	
		**Average ± standard deviation**	**Variation coefficient**	**Average ± standard deviation**	**Variation coefficient**
R. Car. Art.	Diastolic Min. V.	10.33 ± 3.14	30.40%	11.5 ± 2.42	21.04%
	Systolic. Max. V.	72.16 ± 10.4	14.41%	78.5 ± 19.08	24.30%
	Diameter (cm)	0.53 ± 0.06	11.32%	0.55 ± 0.06	10.90%
	PI	1.49 ± 0.19	12.75%	1.47 ± 0.14	9.53%
	RI	0.85 ± 0.06	7.06%	0.85 ± 0.05	5.88%
		**Average ± standard deviation**	**Variation coefficient**	**Average ± standard deviation**	**Variation coefficient**
L. Car. Art.	Diastolic Min. V.	13.33 ± 5.6	42.01%	12.83 ± 3.86	30.08%
	Systolic. Max. V.	71.5 ± 21.6	30.20%	81.16 ± 13.64	16.80%
	Diameter (cm)	0.53 ± 0.06	11.32%	0.56 ± 0.07	12.50%
	PI	1.36 ± 0.28	20.59%	1.45 ± 0.18	12.42%
	RI	0.8 ± 0.12	15.00%	0.84 ± 0.06	7.15%

*R. Car. Art* right carotid artery, *L. Car. Art* left carotid artery, *Min. V* minimum velocity (cm/s), *Max. V* maximum velocity (cm/s), *PI* pulsatility index, *RI* resistance index.

A higher variation coefficient was observed for minimum diastolic velocity, and minimal variation were seen in vessel diameter and resistance index (Table 3).

**Table 3:** Spectral waveform analysis and diameter for normal profile in dogs (n = 12)

	**Average ± standard deviation**	**Variation coefficient**
Minimum Diastolic velocity	12.2 ± 4 cm/s	32.22%
Maximum Systolic velocity	75.8 ± 16 cm/s	21.35%
Diameter	0.544 ± 0.063 cm	11.70%
Pulsatility index	1.44 ± 0.20	13.90%
Resistance index	0.83 ± 0.07	8.44%

DUS vascular studies are being increasingly performed in veterinary medicine and have been proven valuable [1]. However, unlike in humans, detailed investigations of the positive correlations between cerebral disorders and alterations in the common carotid artery of dogs have not been performed in veterinary medicine. The limiting factor observed in the veterinary medical literature is the great variation in sizes and conformations of dog breeds [1]. Therefore, standardization studies must be carried out, if possible, with various breeds that represent these variations.

In the literature, there are values for young beagles [11] that differ from the averages obtained in the present study; previously obtained values include an average common carotid artery systolic peak velocity of 115 ± 17 cm/s and an average final diastolic velocity of 39 ± 7 cm/s. The present study showed a systolic peak velocity of 75.8 ± 16 cm/s and minimum diastolic velocity of 12.2 ± 4 cm/s. This was due to size, corporal mass, fat percentage, age, and anatomical variations in vessel diameter [1].

**Figure 1:**
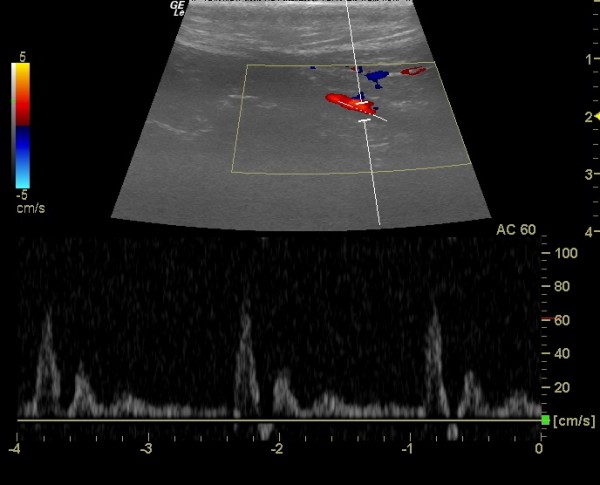
Spectral waveform of the common carotid artery of a dog.

In this study we carried out the standards measurement according of Merritt (2012) as shown in Figure 1, when performing Doppler measurements it is desirable to correct the Doppler angle and shows that in terms of speeds. Therefore, it is necessary to keep the Doppler angle of 60° or less, because small changes in the Doppler angle above 60° result in significant changes in the calculated speed. The lack of accuracy in the measurements results in larger errors in the estimation of speed than similar errors in the angles for measuring levels of impedance, such as resistance index, as these measures are based only on the amplitude ratio between systolic and diastolic.

In 2008, Carvalho et al. described hemodynamic parameters such as resistivity index, allowing comparison of the flow during systole and diastole. These indexes are used to aid in the detection of vascular changes in resistivity in the evaluation of stenosis, thrombosis, or, more commonly, peripheral vessels with increased flow resistance [14-16].

Results of this study allowed us to find an average resistance index of 0.83 ± 0.07, and in the literature on human reports [17] describe an increase in the resistance index of the common carotid artery in patients who developed brain death.

The higher variation coefficient for minimum diastolic velocity and the minimal variation in vessel diameter despite the fact that a clear-cut protocol was followed probably occurred because of the lack of variation in the vessels along their routes and the fact that velocity depends on various factors (measurement site, sample volume reaching the whole vessel diameter, and animal behavior). Great variation in such values has been previously observed [11].

In normal elderly humans, the systolic peak velocity and minimum diastolic velocity are reportedly up to 50% lower than those of younger individuals, which may be explained by vascular complacency. However, no study before ours has attempted to quantify this difference in dogs. Notably, vessel diameter also has a great influence on the systolic peak and must be substantially diminished (about 50%) to lead to hemodynamic alterations [4].

Determining the systolic peak velocity and final diastolic velocity of the common carotid arteries in healthy adult Labrador retrievers may contribute to the establishment of reference values for such hemodynamic variables in dogs of similar size. This will help to develop studies that may correlate the flow in these arteries with neurological alterations (vestibulopathy, stroke, and dementia), thromboembolism, arterial stenosis, and thyroid dysfunction.

## Conclusions

The average systolic peak velocity was 75.8 ± 16 cm/s, average minimum diastolic velocity was 12.2 ± 4 cm/s, average common carotid diameter was 0.545 ± 0.063 cm, and average resistance index was 0.83 ± 0.07. These values may be used as normal parameters for Labrador retriever dogs, thus helping in the diagnosis of neurological disorders associated with alterations in the carotid arteries. Similar studies must be carried out to verify whether the same values can be obtained from dog breeds of different sizes and skull conformations.

## Methods

### Research group

Twelve clinically healthy adult Labrador retriever dogs (6 males and 6 females) aged between 2 and 5 years were used in the present study.

### Consent for participation and ethical approval

All animals were provided by adult owners (dog breeders) and questioned in terms of animal care. The owners then signed a consent form for participation.

The full details of this study were forwarded to the Ethics Committee on Experimentation in the Use of Live Animals of the School of Veterinary Medicine and Animal Science, UNESP, Botucatu, Sao Paulo, Brazil. Ethical approval was granted by this committee (reference no. 222/2011).

### Clinical examination

All animals were subjected to thorough clinical examinations, and only those considered healthy were selected to take part in the experiment (Table 4).

**Table 4:** Animals

	**Females**						**Males**					
**Animal**	**1**	**2**	**3**	**4**	**5**	**6**	**7**	**8**	**9**	**10**	**11**	**12**
**Age (years)**	5	3	5	4	2	4	5	3	4	4	2	3
**Weight (Kg)**	35	35	40	35	40	40	35	40	35	35	40	42

### DUS examination

Animals were preferably placed in a dorsal decubitus position, but some were placed in a lateral decubitus position or even sitting with the maximum neck extension allowed by the animal. The examination site (right and left jugular fossa) was cleaned with detergent followed by alcohol, and acoustic gel was then applied to it.

Examination was performed with the MyLab30 VET (Esaote Healthcare, Brazil) and GE LOGIQe (GE Healthcare, Brazil) ultrasounds system using a linear probe of 5- to 10-MHz frequency, pulse repetition frequency of 4.2 to 4.8 kHz, and approximate gain of 60% to 70%.

**Figure 2:**
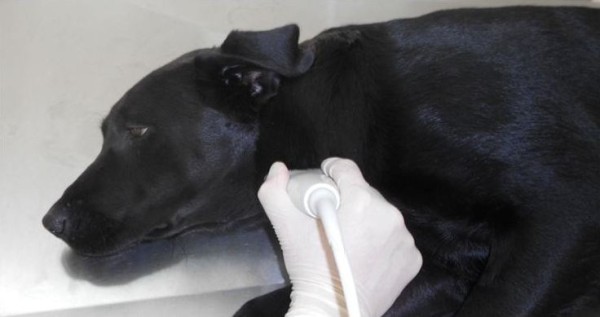
Doppler ultrasonographic examination of the common carotid artery of a dog.

DUS examination of the common carotid artery was conducted on the dogs at rest without sedation (Figure 2).

First, the common carotid arteries were identified by B-scan at the lateral aspects of the trachea, their morphology was evaluated, and their diameters were measured in the transverse plane. Doppler spectral evaluation was performed in the longitudinal plane, and a 52° to 60° constant insonation angle was maintained. The average “sample volume” was 20 mm. The systolic peak velocity and final diastolic velocity of both the right and left common carotid artery were evaluated and measured in cm/s after obtaining at least three complete and clear waves [18-21].

### Data analysis

Data were stored and tabulated. The two-factor ANOVA test was used to compare carotid sides and genders. The average, standard deviation, and variation coefficient were calculated for each variable.

## Competing interests

The authors declare that they have no competing interests.

## Authors’ contributions

DJS and MCTH selected the animals and performed the clinical evaluations. DPD and DJS performed the ultrasonographic examinations. DJS and DPD performed the statistical analysis. DJS, MCTH, and DPD prepared the manuscript. RMA and MJM coordinated the study and revised the manuscript. All authors read and approved the final manuscript.
